# Assessing alternative treatment targets in patients with cystic fibrosis

**DOI:** 10.36416/1806-3756/e20240218

**Published:** 2024-07-29

**Authors:** Rodrigo Cavallazzi, Marcia Pizzichini

**Affiliations:** 1. Division of Pulmonary, Critical Care, and Sleep Disorders. Department of Medicine. University of Louisville, Louisville, KY; 2. Programa de Pós Graduação em Ciências Médicas Universidade Federal de Santa Catarina (UFSC), Florianópolis,SC

Cystic fibrosis is an autosomal recessive genetic disease that involves multiple organ systems. The cystic fibrosis transmembrane conductance regulator (CFTR) gene on chromosome 7 encodes the CFTR protein, an ion channel responsible for transporting chloride ions from inside the cells to the airway lumen. The presence of two pathogenic variants of the CFTR leads to malfunction of the channel with consequent viscous secretions mainly affecting the gut and lungs. There are over 2000 mutations in the CFTR gene,^1^ with 700 of these being disease-causing variants.^2^ The most common variant is the *F508del*,^3^ which leads to phenylalanine deletion at position 508 and consequent misfolding and degradation of the CFTR protein.^2^ The frequency of *F508del* variant in people with cystic fibrosis varies globally. For example, it is present in 85% of the population with cystic fibrosis in areas with predominant European ancestry^3^ and 38% in a cohort from South India.^4^ In Brazil, the *F508del* variant is found in 52.8% of individuals with cystic fibrosis, with 24.5% being homozygous for the *F508del* variant and 28.8% being heterozygous.^5^

An estimate based on data from 5 Brazilian states from the South and Southeast showed an incidence of cystic fibrosis homozygotes of 1 in 20,202 (95% CI 8,651-47,179). The incidence ranged from 1 in 1,587 (95% CI 504-5005) in Rio Grande do Sul to 1 in 32,258 (95% CI 3,281-318,549) in Sao Paulo.^6^ In Brazil, the median age of individuals with cystic fibrosis is 10.8 years,^5^ ^)^ contrasting with 21.9 years in the United States,^3^ ^)^ with adults comprising 25% of the population affected.^5^ Among Brazilian individuals with cystic fibrosis, the racial distribution is as follows: white (69%), mixed-race (24%), black (6%), Asian (1%), and Indigenous (1%). However, it is important to note that these data are not based on self-declared race.^5^

The diagnosis of cystic fibrosis is established when there is clinical suspicion (e.g. a positive newborn screening test or clinical symptoms or family history) plus a positive sweat chloride test (≥60 mmol/L) or two cystic fibrosis-causing mutations when the sweat chloride test is in the intermediate range (30 - 59 mmol/L).^7^ All patients with a diagnosis of cystic fibrosis should undergo genetic testing.^8^ Where newborn screening for cystic fibrosis is widely implemented, most cases are detected early in the asymptomatic phase by screening.^3^ Clinically, patients with cystic fibrosis can present in a variety of ways. Approximately 15% of the patients present with meconium ileus as neonates.^9^ In those aged ≥ 1 year upon diagnosis, almost half of the patients have respiratory symptoms. Other common presenting manifestations include nasal polyps and sinus disease, steatorrhea, infertility, malnutrition, failure to thrive, and digital clubbing. Over time, these patients develop bronchiectasis, obstructive lung disease, and recurrent respiratory infections, mainly by *Staphylococcus aureus* and *Pseudomonas aeruginosa*.^3^ Most deaths are due to respiratory or cardiorespiratory failure.^3^

The survival of patients with cystic fibrosis has been steadily improving. In the United States, the predicted survival age for individuals with cystic fibrosis is currently estimated at 68.2 years.^3^ In Brazil, analysis based on death certificate reports has shown a notable increase in the median age of individuals who died from cystic fibrosis over time,^10^ which is likely attributable to advances in cystic fibrosis management and the establishment of cystic fibrosis centers.^11^ However, substantial regional healthcare disparities exist in the diagnosis and management of cystic fibrosis patients in Brazil^12^ Continued improvement in patient outcomes require addressing these disparities, ensuring universal and effective access to newborn screening, reliable sweat tests,^12^ and the expansion of multidisciplinary cystic fibrosis centers.^11^

A revolution in the treatment of patients with cystic fibrosis has been the introduction of the CFTR modulators. These medications act as potentiators, by improving the function of the CFTR, or as correctors, by increasing the amount of CFTR in the airway epithelia. CFTR modulators improve respiratory symptoms, lung function, and nutritional status; reduce the frequency of exacerbations; and lower sweat chloride concentraton.^2^ In late 2020, ivacaftor was included into the *Sistema Único de Saúde* (Brazil’s public health care system) for patients aged 6 years and with gating mutations.^13^ In September 2023, the Brazilian Ministry of Health announced the inclusion of elexacaftor-tezacaftor-ivacaftor for patients aged 6 years and older with at least one *F508del* mutation in the *CFTR* gene. This decision is expected to benefit an estimated 1,700 eligible patients.^14^

Despite significant advancements obtained by newborn screening, diagnostic tests, treatment of complications, multidisciplinary care, cystic fibrosis centers, and CFTR modulators, cystic fibrosis continues to be a disease with a challenging prognosis. Alternative treatment targets have the potential to further improve the outcomes of these patients. One of them is TMEM16A, a Ca2+-activated chloride channel present in airway epithelial and smooth cells. Basic research has shown that TMEM16A mediates mucus secretion and can be upregulated by cytokines in human airway epithelial cells. Additionally, inhibitors of TMEM16A, such as niclosamide, and benzbromarone, lead to decreased airway mucus production although this effect could also be due to inhibition of other proteins.^16^ Both inhibitors and activators of TMEM16A may prove to have a role in the treatment of inflammatory airway diseases, including cystic fibrosis. An intriguing finding is that is that cells expressing F508 del have impaired expression of TMEM16A by its regulator, CLCA1. This indicates an interdependence between TMEM16A and CFTR.^16^ This also brings the possibility that the effect of inhibitors and activators of TMEM16A may be influenced by CFTR modulators.

In this edition of the *Jornal Brasileiro de Pneumologia*, Friedrich et al.^15^ conducted a single center, single arm pilot trial assessing the safety of benzbromarone in children > 6 years with confirmed cystic fibrosis. In addition to the standard treatment for cystic fibrosis, which at the time did not include CFTR modulators presumably due to their unavailability, patients were administered 100 mg of oral benzbromarone once daily for a duration of 90 days. The primary outcome was the safety of benzbromarone in these patients. One patient developed a 3 kg weight loss, which improved after the medication was discontinued. One patient experienced a pulmonary exacerbation during the study period. One patient chose to withdraw from the study before its completion. The FEV_1_ in percentage of predicted increased by 8% although the difference did not quite reach statistical difference.

This proof-of-concept study explores an alternative treatment approach for patients with cystic fibrosis, based on robust foundational research.^16^ The tested medication, the uricosuric agent benzbromarone, is cost-effective and has been used for a different condition for decades. However, the study’s small sample size precludes definitive conclusions regarding the medication’s safety in pediatric patients with cystic fibrosis. Future studies, ideally multicenter and placebo-controlled with safety outcomes adjudication, are needed to establish benzbromarone’s safety in this population. Nevertheless, this study marks an initial step toward understanding the medication’s effects and safety in pediatric patients with cystic fibrosis, for which the authors deserve commendation.
